# Immature granulocyte percentage for prediction of sepsis in severe burn patients: a machine leaning-based approach

**DOI:** 10.1186/s12879-021-06971-2

**Published:** 2021-12-16

**Authors:** Kibum Jeon, Nuri Lee, Seri Jeong, Min-Jeong Park, Wonkeun Song

**Affiliations:** 1 Department of Laboratory Medicine, Hallym University Hangang Sacred Heart Hospital, 12 Beodeunaru-ro 7-gil, Yeongdeungpo-gu, Seoul, 150719 South Korea; 2 Department of Laboratory Medicine, Hallym University Kangnam Sacred Heart Hospital, Hallym University College of Medicine, 22, Singil-ro 1, Yeongdeungpo-gu, Seoul, 07440 South Korea

**Keywords:** Sepsis, Immature granulocytes, Machine learning, Severe burn

## Abstract

**Background:**

Of the existing sepsis markers, immature granulocytes (IG) most frequently reflect the presence of an infection. The importance of IG as an early predictor of sepsis and bacteremia is evaluated differently for each study. This study aimed to evaluate the effectiveness of the Sysmex XN series’ IG% as an independent prognostic indicator of sepsis using machine learning.

**Methods:**

A total of 2465 IG% results from 117 severe burn patients in the intensive care unit of one institution were retrospectively analyzed. We evaluated the IG% for sepsis using the receiver operating characteristic, logistic regression, and partial dependence plot analyses. Clinical characteristics and other laboratory markers associated with sepsis, including WBC, procalcitonin, and C-reactive protein, were compared with the IG% values.

**Results:**

Twenty-six of the 117 patients were diagnosed with sepsis. The median IG% value was 2.6% (95% CI: 1.4–3.1). The area under the receiver operating characteristic curve was 0.77 (95% CI: 0.78–0.84) and the optimal cut-off value was 3%, with a sensitivity of 76.9% and specificity of 68.1%. The partial dependence plot of IG% on predicting sepsis showed that an IG% < 4% had low predictability, but increased thereafter. The interaction plot of IG% and C-reactive protein showed an increase in sepsis probability at an IG% of 6% and C-reactive protein of 160 mg/L.

**Conclusions:**

IG% is moderately useful for predicting sepsis. However, since it can be determined from routine laboratory test results and requires no additional intervention or cost, it could be particularly useful as an auxiliary marker.

**Supplementary Information:**

The online version contains supplementary material available at 10.1186/s12879-021-06971-2.

## Background

Burns are complex traumatic injuries that require appropriate treatment, including surgical intervention in the acute phase. Since the skin is the first barrier to infection, burn patients are constantly exposed to inflammatory mediators while the wound remains exposed. As a result, severe burn patients often develop persistent systemic inflammatory response syndrome (SIRS), which can lead to sepsis [1].

Sepsis, which occurs when SIRS is accompanied by an infection, is common in burn patients. This is due to the dysregulation of the host’s response to infection and can be a major risk factor for mortality. The early diagnosis of sepsis before progression to organ dysfunction has a decisive effect on the clinical course and outcomes of patients with severe sepsis [2]. However, since sepsis is a clinical syndrome involving many heterogeneous conditions and not a final diagnosis, there is no definitive model for the diagnosis of sepsis [3]. Furthermore, patients with severe burns have persistent tachycardia, tachypnea, and fever due to SIRS, making it difficult to predict sepsis based on clinical signs. Thus, biomarkers are of high clinical importance for predicting sepsis in burn patients.

Immature granulocytes (IG) are recently-produced granulocytes that have been released into the circulation. While IG are normally absent from peripheral blood, they increase in conditions such as bacterial sepsis, inflammation, trauma, cancer, steroid therapy, and myeloproliferative diseases [4, 5]. Previous studies have shown that the IG percentage (IG%) is clinically significant for the early detection of bacterial infections or in predicting the risk of sepsis in the intensive care unit. However, most studies have been conducted on pediatric patients, and few have studied adult burn patients [6–8]. In addition, the importance of IG as an early predictor of sepsis has been evaluated differently in several studies. Differences in patient groups or confounding factors could contribute to these differences [9, 10]. Therefore, it is necessary to clarify whether the IG% could be an independent indicator of prognosis compared to existing sepsis indicators, especially for severe burn patients.

In this study, we evaluated the effectiveness of the IG% as an independent predictor of sepsis in patients with severe burns using the IG index of the Sysmex XN (Sysmex, Kobe, Japan), which can objectively calculate the IG%. Moreover, as machine learning has recently received attention as an effective prediction tool that may improve the accuracy of predictions, we used a machine learning-based prediction model.

## Methods

### Patients

This study included 117 patients treated in the burn intensive care unit (BICU) at the Burn Center, Hallym University Hangang Sacred Hospital, Seoul, Republic of Korea between July and December 2017. Data collected for each patient were as follows: age, sex, percentage of total body surface area burned (%TBSA), cause of burn injury, and presence of inhalation injury. All patients with burns were newly diagnosed, with no history of underlying disease. The study was approved by the institutional review board of the Hallym University Hangang Sacred Hospital (IRB no. HG2018-069), which waived the requirement for written informed consent owing to the observational and anonymized nature of the study. The study was performed in accordance with the guidelines of the Declaration of Helsinki.

### Diagnosis of sepsis in burn patients

We classified a sepsis group according to the diagnostic criteria for sepsis in burn patients of the American Burn Society (Table 1) [11]. Patients who did not belong to this group were classified into the non-sepsis group. The date of diagnosis of sepsis was designated as the first day that satisfies the diagnostic criteria in Table 1 during inpatient treatment in BICU.

**Table 1:** Diagnostic criteria for sepsis in burn patients

Culture positive infection and at least three of the following:
1. Fever > 39 °C
2. Hypothermia (< 36.5 °C)
3. Progressive tachycardia (> 110 beats per min)
4. Progressive tachypnea (> 25 breaths per minute not ventilated or minute ventilation > 12 L/min ventilated)
5. Thrombocytopenia (< 100,000/μl)
6. Hyperglycemia, in the absence of preexisting diabetes mellitus (untreated plasma glucose > 200 mg/dl or > 7 units of insulin/h intravenous drip or significant resistance to insulin, > 25% increase in insulin requirement over 24 h);
7. Inability to continue enteral feedings > 24 h (abdominal distension or high gastric residuals, residuals two times feeding rate or uncontrollable diarrhea, > 2500 ml/day)

### Measurement of IG and biomarkers associated with sepsis

**Fig. 1:**
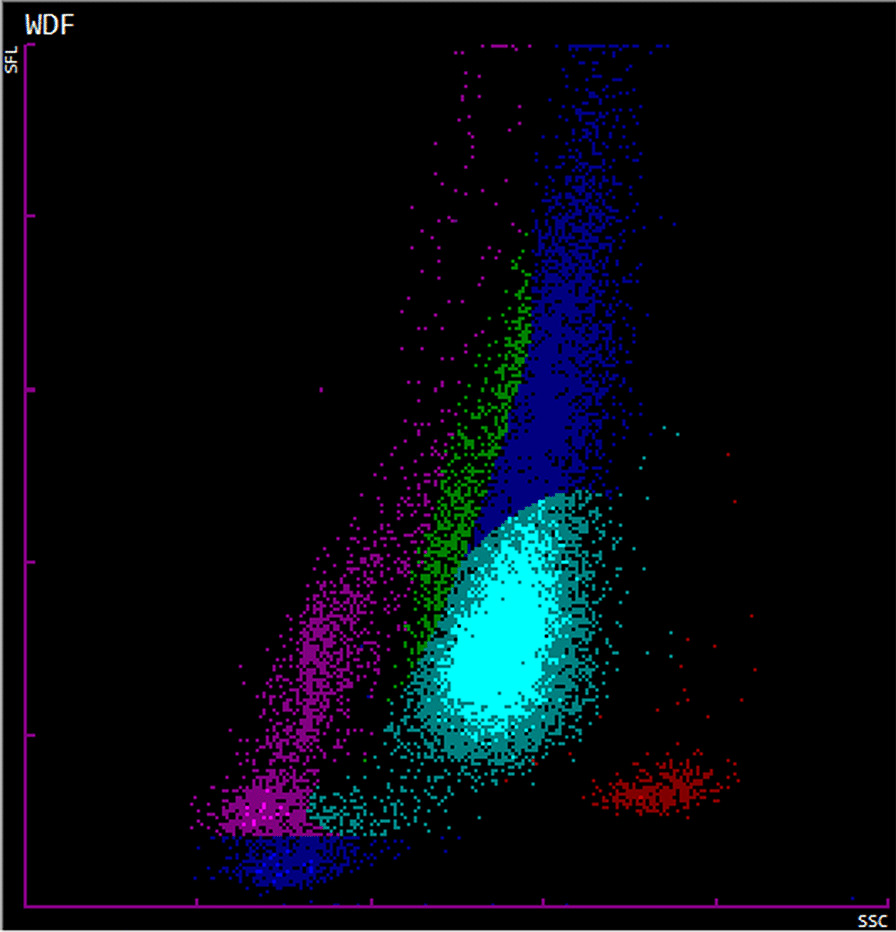
White cell differential channel (WDF) scattergram of a burn patient with sepsis, IG% of 17.1%. The WDF channel differentiates and counts lymphocytes (purple), monocytes (green), eosinophils (red), mature neutrophils (cyan), Immature Granulocytes (blue). Signal fluorescence intensity (SFL) was used to separate cells according to the DNA/RNA content and determine the immaturity and activation of the examined cells (y axis). Forward scattering (FSC, z axis) and side scattering (SSC, x axis) indicate cell size and the complexity of the intracellular structure, respectively

For the complete blood count, which included the IG% and IG count, were analyzed by K3-EDTA (BD, Franklin Lakes, NJ) anticoagulated venous whole blood samples using the XN-1000 automated hematology analyzer (Sysmex, Kobe, Japan). For measure IG, the white cell differential channel (WDF) was used after applying a specific lysing agent (Lysercell WDF), and differentiation was determined according to granularity (side scatter) and nucleic acid content (side fluorescence by the Fluorocell WDF reagent). The IG cluster was found right above the neutrophil cluster in the scatter/fluorescence of the biparametric histogram side (Fig. 1). The IG% was calculated using the following formula: (particle count in IG zone/particle count in WBC zone) × 100. The IG% included promyelocytes, myelocytes, and metamyelocytes, while blasts and band cells were not included [12].

Biomarkers associated with sepsis, including procalcitonin, C-reactive protein (CRP), and lactate, were compared to the IG%. All samples were analyzed using SST-II (BD, Franklin Lakes, NJ, USA) serum samples. Procalcitonin and CRP levels were measured using a Cobas c702 (Roche Diagnostics, Mannheim, Germany), and lactate was analyzed using an Ultra-M Analyzer (Nova Biomedical, Waltham, MA, USA). The highest IG% and biomarker values before or at the same time point of sepsis for each patient were used.

### Statistical analyses

Statistical analyses were performed using the SPSS statistics program version 24 (IBM Corporation, New York, NY) and MedCalc version 18 (MedCalc Software, Mariakerke, Belgium). Quantitative variables are presented as the median and interquartile range (IQR: 25th–75th percentiles). The Mann–Whitney test was applied to compare nonparametric quantitative variables between the two groups, whereas the Kruskal–Wallis test was used to compare more than two groups. The chi-square test was used to compare the distribution of the two groups.

Receiver operating characteristic (ROC) curves were used to compare the IG% and other parameters between the sepsis and non-sepsis patient groups. The IG% diagnostic cut-off value with the best combined sensitivity and specificity was also determined. The area under the curve (AUC) and 95% confidence interval (CI) were calculated for each plot.

A logistic regression analysis was performed to evaluate the predictive value of the IG% for sepsis. Univariate and multivariate regression analyses were performed for the biomarkers and risk factors. Statistical significance was set at *P* < 0.05.

### Machine learning approach

The machine learning interpretability techniques were analyzed using the R-based pdp package (version 3.6.3). Partial dependence plots (PDP) and individual conditional expectations (ICE) are used to interpret and visualize the partial dependence of the probability of developing an outcome (sepsis) on a predictor (IG%, biomarkers, and risk factors). Both single-predictor and multi-predictor PDP were performed for this study. For the multi-predictor PDP, the grid.arrange function was used to display a color map for level plots. ICE curves were displayed on the same plot with the average PDP using the ggplot2 package and centered on the zero base. The schematic diagram of machine learning process is provided in the Additional file 1: Fig. S1.

## Results

### Patient characteristics in the sepsis and non-sepsis groups

The demographic characteristics of the 117 patients, 26 of which were in the sepsis group (22.2%) and 91 in the non-sepsis group (77.8%), are summarized in Table 2. The %TBSA, presence of inhalation burns, length of stay in the BICU, death rates and positive rate of blood culture were significantly different between the two groups (P < 0.001).

**Table 2:** Demographic and laboratory characteristics of the patients

	All (N = 117)	Sepsis (N = 26)	Non-sepsis (N = 91)
Age, year	52 (41.5–60.0)	49.5 (28.0–56.0)	52.0 (43.2–60.0)
Gender (M:F)	92:25	22:4	70:21
%TBSA	26.0 (15.0–43.5)	57.5 (33.0–60.0)*	22.0 (12.0–31.0)
Inhalation burn (Y:N)	46:71	18:8*	28:63
Full thickness burn (Y:N)	62:54	16:10	46:44
Mode Flame/Electric/Scalding/Chemical/other	68/20/18/8/3	21/0/2/3/0	47/20/16/5/3
SAP-3 score	32.0 (27.0–38.5)	33.0 (30.0–39.0)	31.5 (25.0–37.0)
Day of ICU stay	9.0 (4.0–29.5)	26.0 (15.5–52.0)*	7.0 (3.3–20.8)
Death (Y:N)	25:93	11:16*	14:77
IG%	2.6 (0.8–5.2)	6.4 (3.1–11.6)*	1.4 (0.6–4.4)
IG count (× 10^9^/ L)	0.34 (0.07–0.85)	0.64 (0.30–2.02)*	0.21 (0.06–0.63)
WBC (× 10^9^/ L)	11.8 (7.8–15.8)	10.4 (4.5–15.2)	12.0 (8.4–16.0)
Absolute neutrophil count (× 10^9^/ L)	9.0 (5.4–12.0)	8.1 (3.8–11.7)	9.4 (5.7–12.2)
Procalcitonin (μg/L)	1.18 (0.52–4.19)	1.72 (0.69–5.47)	0.82 (0.31–4.17)
CRP (mg/L)	103.6 (41.7–173.6)	176.5 (148.6–221.8)*	77.8 (33.2–143.6)
Lactate (mmol/L)	3.3 (2.2–5.1)	4.9 (3.5–6.4)*	2.8 (2.2–4.7)
Positive blood Culture (%)	39 (33.3)	26 (100)*	13 (14.3)
Total organisms detected number (%)	65	47	18
*Acinetobacter baumannii*	24 (36.9)	16 (34.0)	*8 (44.4)*
CRAB	6 (9.2)	4 (8.5)	*2 (11.1)*
*Pseudomonas aeruginosa*	12 (18.5)	9 (19.1)	*3 (16.7)*
CRPA	4 (6.2)	3 (6.4)	*1 (5.6)*
*Enterococcus spp.*	5 (7.7)	4 (8.5)	*1 (5.6)*
*Klebsiella pneumoniae*	4 (6.2)	4 (8.5)	*0*
MRSA	3 (4.6)	2 (4.3)	*1 (5.6)*
*Proteus mirabilis*	2 (3.1)	2 (4.3)	*0*
*Escherichia coli*	2 (3.1)	1 (2.1)	*1 (5.6)*
Others	3 (4.6)	2 (4.3)	*1 (5.6)*
Antibiotics use	93 (79.5)	26 (100)	67 (73.6)

Continuous data are quoted as median values with interquartile range

*P < 0.05, Data are compared using a Mann–Whitney or Chi-square test between “sepsis” and “non- sepsis”

*IG* immature granulocyte, *TBSA* total body surface area, *ICU* intensive care unit, *SAPS-3* simplified acute physiology score-3, *WBC* white blood cell, *CRP* c-reactive protein, *CRAB* Carbapenem-resistant *Acinetobacter baumannii*, *CRPA* Carbapenem-resistant *Pseudomonas aeruginosa*, *MRSA* Methicillin-resistant *Staphylococcus aureus*

### Utility of biomarkers for predicting sepsis

#### WBC and absolute neutrophil count

The median [IQR] WBC count in the sepsis group and non-sepsis group was 10.4 × 10^9^/L [4.5–15.2 × 10^9^/L] and 12.0 × 10^9^/L [8.4–16.0 × 10^9^/L], respectively. The absolute neutrophil count was 8.1 × 10^9^/L [3.8–11.7 × 10^9^/L] and 9.4 × 10^9^/L [5.7–12.2 × 10^9^/L], respectively. While both were higher in the non-sepsis group, the differences were not statistically significant.

#### IG% and IG count

In the sepsis group, the median [IQR] IG% value was 6.4% [3.1–11.6%], which was significantly higher than that in the non-sepsis group (1.4% [0.6–4.4%], *P* < 0.001). The IG count was also significantly higher in the sepsis group (0.64 × 10^9^/L [0.30–2.02 × 10^9^/L], *P* < 0.001) than in the non-sepsis group (0.21 × 10^9^/L [0.06–0.63 × 10^9^/L]).

#### CRP, lactate, and procalcitonin

CRP and lactate levels [IQR] were higher in the sepsis group than in the non-sepsis group (CRP: 176.5 mg/L [148.6–221.8] vs. 77.8 mg/L [33.2–143.6] and lactate: 4.9 mmol/L [3.5–6.4] vs. 2.8 mmol/L [2.2–4.7], respectively, all *P* < 0.001). Procalcitonin levels were also higher in the sepsis group than in the non-sepsis group, but the difference was not statistically significant.

### Correlation between WBC count and IG%

**Fig. 2:**
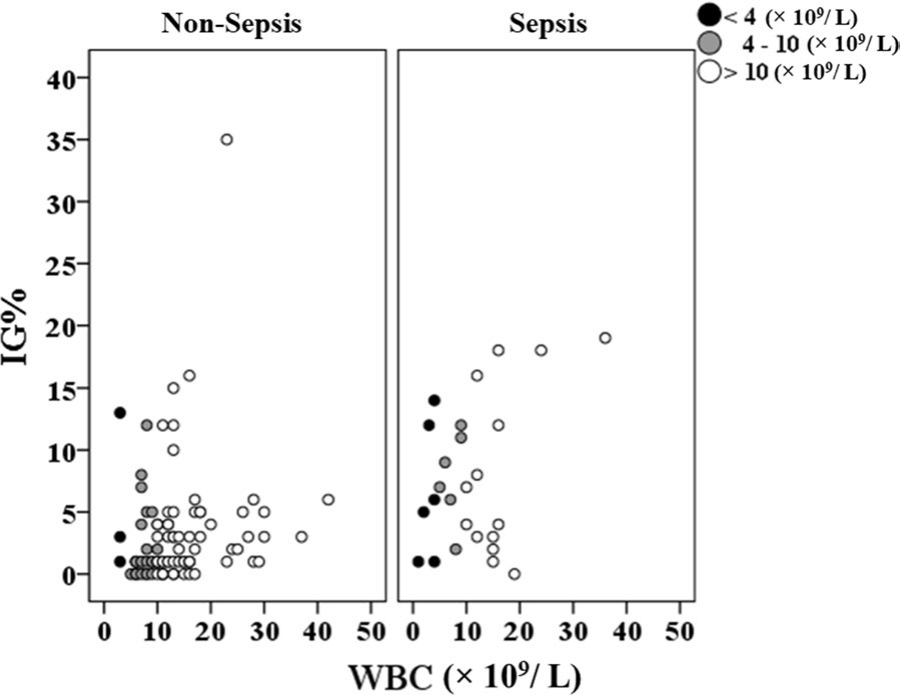
Distribution of IG% as related to the WBC count in the sepsis and non-sepsis groups. The patient groups were divided into 3 subgroups based on the WBC count (< 4 × 10^9^/L; 4–10 × 10^9^/L; and > 10 × 10^9^/L)

The IG% in the sepsis group was also higher overall than that in the non-sepsis group, both for patients with a WBC count < 4.0 × 10^9^/L and those with a normal WBC count (Fig. 2). For patients with a WBC count < 4.0 × 10^9^/L, 4.0–10.0 × 10^9^/L, and > 10.0 × 10^9^/L in the sepsis group, the IG% was 5.5% (1.0–12.0%), 8.0% (6.0–11.0%), and 5.5% (3.0–16.0), respectively (*P* = 0.823). For patients with a WBC count < 4.0 × 10^9^/L, 4.0–10.0 × 10^9^/L, and > 10.0 × 10^9^/L in the non-sepsis group, the IG% was 3.0% (1.0–5.10%), 1.0% (0.0–2.0%), and 3.0% (2.0–8.0%), respectively (*P* = 0.065).

### ROC curve analysis

**Fig. 3:**
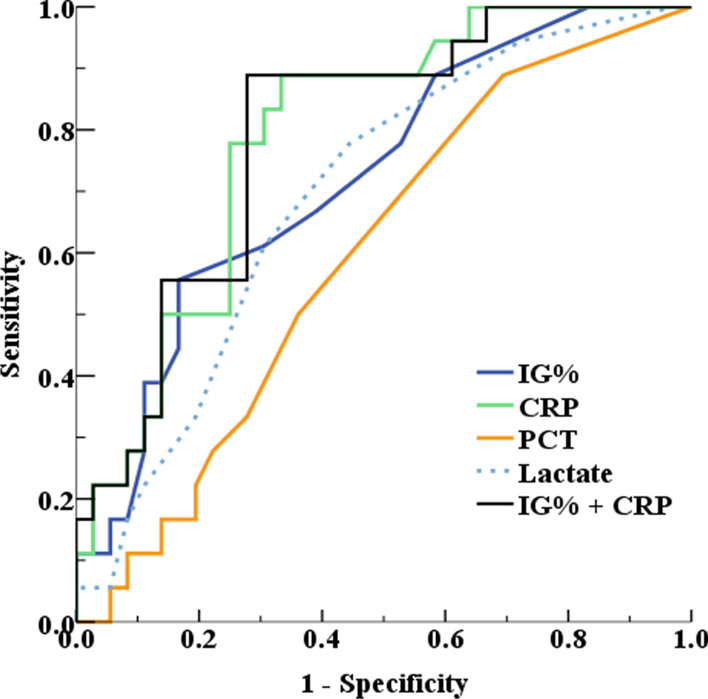
Receiver operator characteristic (ROC) curve to evaluate the biomarkers for diagnosis of sepsis in burns. *IG%* immature granulocyte percentage, *CRP* C-reactive protein, *PCT* procalcitonin

The ROC curve analysis was performed to evaluate the differences in the sensitivities and specificities of the IG% and biomarkers in the sepsis and non-sepsis groups. The AUC was highest for CRP (0.82, 95% CI: 0.74–0.89), followed by IG% (0.77, 95% CI: 0.68–0.84), lactate (0.73, 95% CI: 0.64–0.81), and procalcitonin (0.610, 95% CI: 0.46–0.74). The AUC of combined IG% and CRP showed higher AUC (0.85, 95% CI: 0.78–0.92), compared to that of CRP or IG% alone. The optimal cut-off value for the IG% was 3%, with a sensitivity of 76.9% (95% CI: 56.4–91.0) and specificity of 68.1% (95% CI: 57.5–77.5) (Fig. 3, Table 3).

**Table 3:** Receiver operating characteristic analysis of IG%, PCT, CRP, and lactate for discriminate the sepsis

Biomarker	Sepsis			
	AUC (95% CI)	Optimal cutoff	Sensitivity (95% CI)	Specificity (95% CI)
IG%	0.77 (0.68, 0.84)	3.0	76.9 (56.4, 91.0)	68.1 (57.5, 77.5)
CRP	0.82 (0.74, 0.89)	15.3	76.0 (54.9, 90.6)	79.3 (69.3, 87.3)
Lactate	0.73 (0.64, 0.81)	3.1	88.5 (69.8, 97.6)	59.1 (48.1, 69.5)
Procalcitonin	0.61 (0.46, 0.74)	0.32	94.4 (72.7, 99.9)	30.6 (16.3, 48.1)

### Logistic regression analysis

We analyzed the predictive power of the IG% and other biomarkers for the diagnosis of sepsis using logistic regression analysis. The odds ratio [95% CI] for IG% (1.15 [1.05–1.25]), CRP (1.01 [1.01–1.02]), and lactate (1.22 [1.07–1.39]) were found to be significant (Table 4). We further analyzed the significant variables using multivariate logistic regression with known risk factors (age, %TBSA, and presence of inhalation burns), which revealed that the odds ratio [95% CI] for IG% (1.09 [1.00–1.08]) and CRP (1.02 [1.03–1.08]) were significantly associated with sepsis.

**Table 4:** Logistic regression analysis for biomarkers predicting sepsis

Variables	Univariate analysis, OR (95% CI)	*P*-value	Multivariate analysis*, OR (95% CI)	*P*-value
Age (year)	0.98 (0.96–1.01)	0.263		
Inhalation burn	2.02 (1.19–3.41)	0.009		
TSBA%	1.05 (1.02–1.07)	< 0.001		
IG%	1.15 (1.05–1.25)	0.002	1.09 (1.00–1.18)	0.043
Procalcitonin (μg/L)	0.99 (0.95–1.03)	0.666		
CRP (mg/L)	1.02 (1.01–1.02)	< 0.01	1.02 (1.03–1.08)	< 0.001
lactate (mmol/L)	1.16 (1.01–1.33)	0.032	1.06 (0.88–1.26)	0.542

*Multivariate analysis adjusted for age, TBSA, and inhalation injury

### Machine learning application for evaluating the IG%

#### PDP for sepsis prediction

**Fig. 4:**
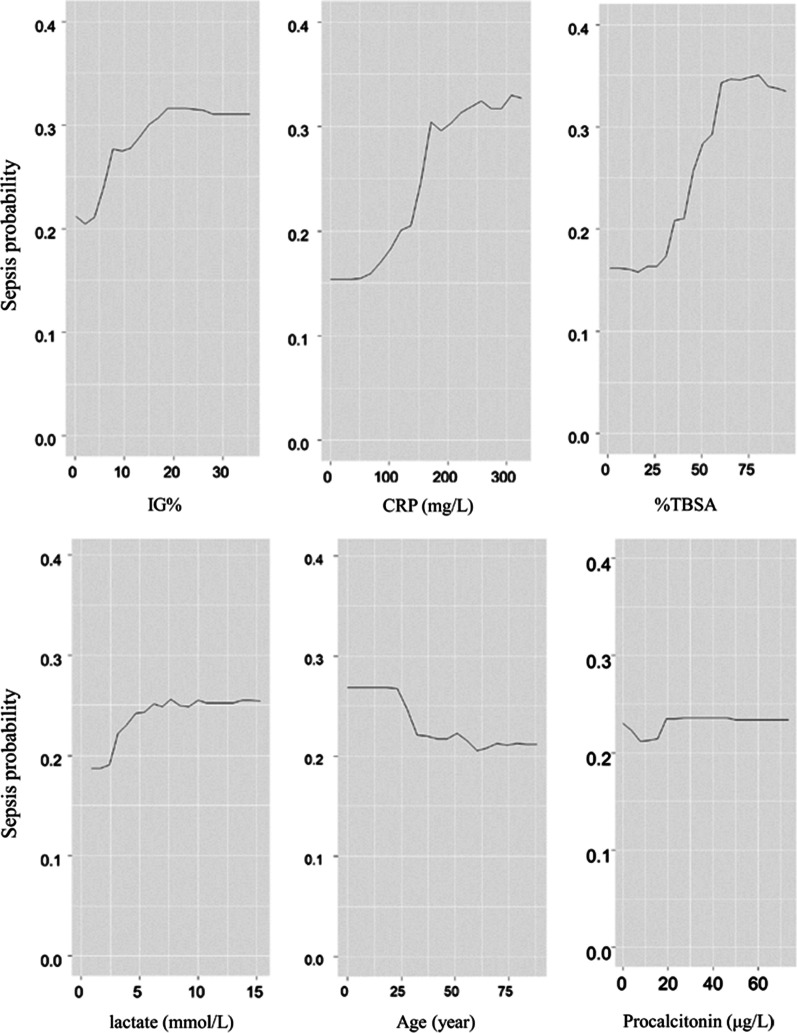
Partial dependent plots (PDP) for sepsis prediction. For IG%, the PDP showed that the probability was low until 4%, and increased rapidly after. In CRP and TBSA, probability plot increased sharply above 140 mg/L and 30%, respectively. Lactate’s increased when above 2.5 mmol/L but showed less change. Sepsis probability decreased with age, age between 20 and 25 years-old had higher predicted sepsis risk. For procalcitonin, the sepsis probability does not change significantly. *IG%* immature granulocyte percentage, *CRP* C-reactive protein, *%TBSA* total body surface area percentage

Figure 4 shows the single-predictor PDP of each variable’s predictive value for predicting sepsis, including the IG%, CRP, %TBSA, lactate, age, and procalcitonin. In this visual plot, the probability of developing sepsis was found to increase significantly as the values of IG%, CRP, and %TBSA increased. The PDP showed that an IG% < 4% had a low probability of sepsis that increased thereafter. For CRP and %TBSA, the probability plot increased sharply above 140 mg/L and 30%, respectively. The probability of developing sepsis increased as lactate levels exceeded 2.5 mmol/L, but the change was not as sharp. Sepsis probability decreased with age such that those aged 20–25 years had the highest predicted sepsis risk. For procalcitonin, the probability of sepsis did not change significantly.

#### ICE plot for sepsis probability according to IG%

**Fig. 5:**
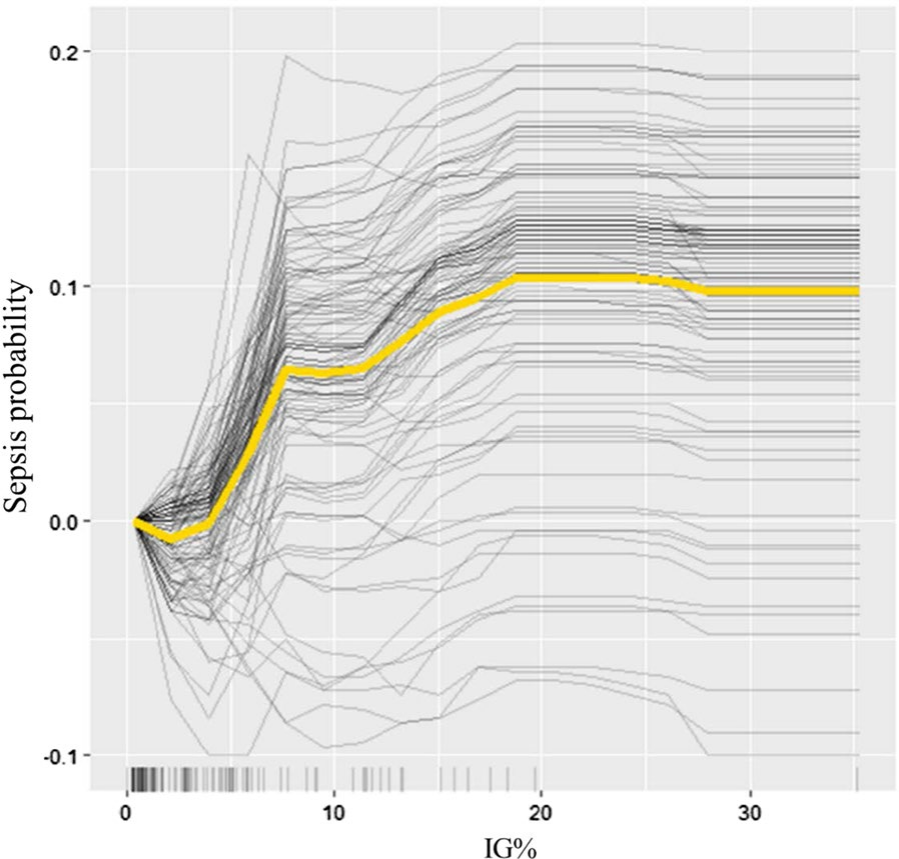
Individual conditional expectation (ICE) plot for sepsis probability according to IG%. Each line represents one patient. Centered line (Yellow) represents the mean of lines, started with a sepsis probability of zero. In most patients, sepsis probability increased as IG% increased. The prediction unchanged until the IG% of 4 and increases thereafter

On the ICE plot for IG%, the centered line (mean), starting at 0 for sepsis probability, increased rapidly after an IG% of 4%. The other lines (individual patients) showed similar patterns, with most patients showing an increase in predicted sepsis probability with increasing IG% (Fig. 5).

#### Multi-predictor PDP of sepsis probability

**Fig. 6:**
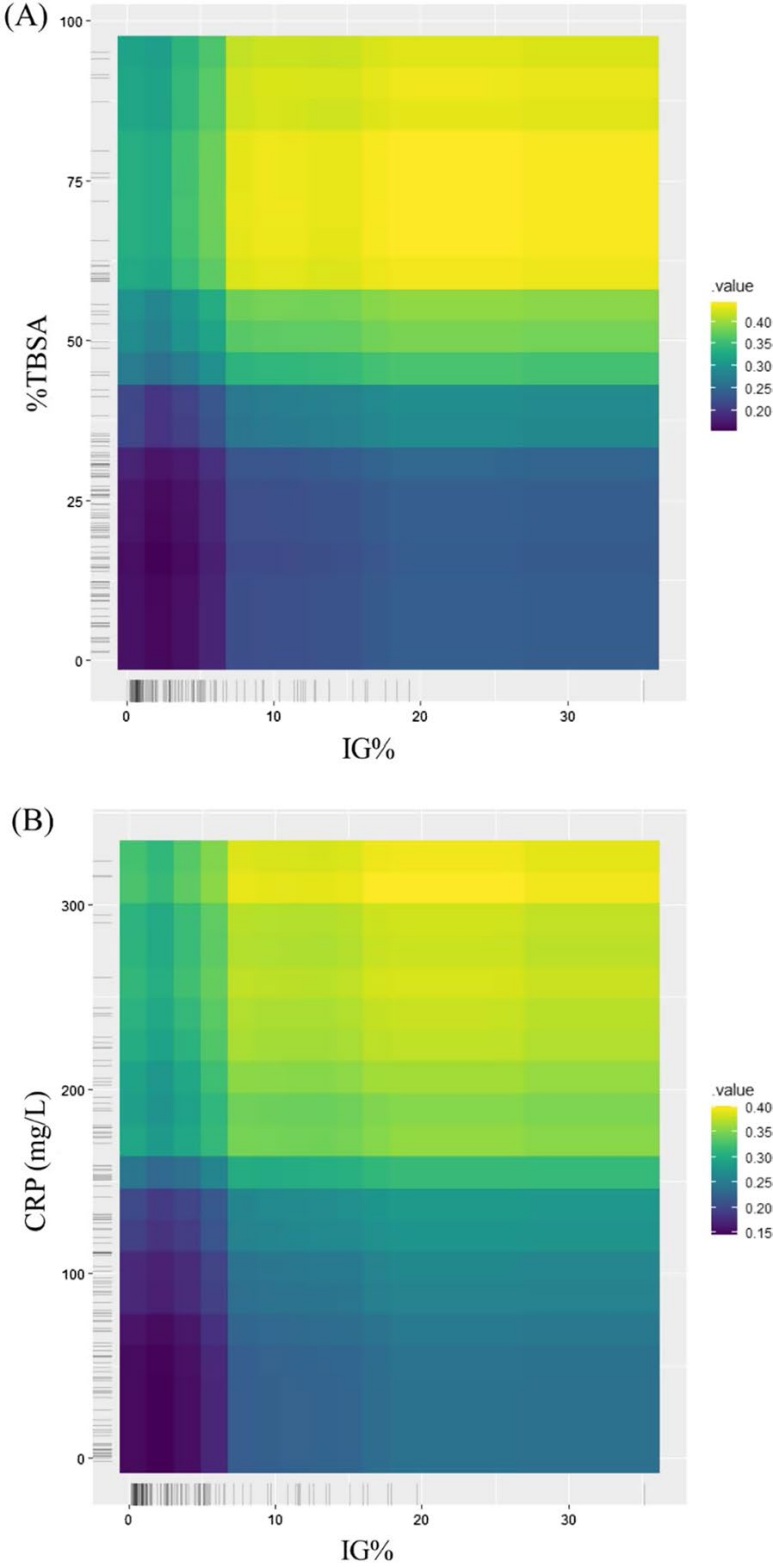
Multi-predictor PDP of sepsis probability (**A**) IG% and total body surface areas (TBSA) of burns (**B**) IG% and CRP. Brighter color shows the increase in sepsis probability. For IG% above 6, patient with TBSA above 60% and CRP over 160 mg/L have higher predicted sepsis risk

The multi-predictor PDP of sepsis probability and the interaction between IG% and %TBSA and between IG% and CRP are described in Fig. 6A, B, respectively. The plot shows an increase in sepsis probability at an IG% of 5% or 6%. For an IG% above 6%, patients with > 60% TBSA or a CRP > 160 mg/L had a higher predicted sepsis risk that was near to or greater than 0.4, which was higher than the predictability of any single parameter alone.

## Discussion

In this study, IG% showed moderate predictability for sepsis in patients with severe burns. There was no significant difference in WBC count or absolute neutrophil count between the sepsis and non-sepsis groups; however, while the non-sepsis group showed a slightly higher IG count, the IG% was significantly higher in the sepsis group. CRP and lactate levels were higher in the sepsis group than in the non-sepsis group; however, the differences in the percentages were not as large as those for the IG%. The usefulness of the IG% was also demonstrated using the ROC curve and logistic regression analysis. The IG% had the second-highest AUC after CRP, indicating that this parameter showed good discrimination between the two groups. Logistic regression analysis showed that the IG% was significantly associated with the development of sepsis.

Our results are consistent with those of previous studies. Porizka et al. [13] reported that IG% was significantly higher in cardiac surgical patients with sepsis than in those with non-infective SIRS, indicating that IG% is a helpful marker with a moderate ability to discriminate between sepsis and non-sepsis. Hampson et al. [14] showed that the IG count was elevated in septic patients with burn injuries. In our study, moreover, the relationship between IG%, other biomarkers, and known risk factors for sepsis probability were analyzed using machine learning.

Machine learning algorithms have demonstrated the ability to automate the interpretation and analysis of laboratory data in a variety of fields [15] and have also been adapted in medical research in recent years. We used PDP and ICE plots to visualize the relationship between the predictors and sepsis predictability. PDP are low-dimensional graphical renderings of the prediction function and are especially useful for visualizing relationships discovered by complex machine learning algorithms. This also allows for any number of predictors to be assessed, and a multivariate display can thus be obtained. The PDP is the average of the lines of the ICE plot. ICE plots display one line per individual patients that shows how the patient’s prediction changes when IG% changes [16, 17].

The results of the single-predictor PDP showed that the IG% significantly increased the probability of sepsis, after the %TBSA and CRP. The sepsis probability was low until an IG% of 3% was reached but increased rapidly above 0.3 when the IG% exceeded 4%. This was consistent with the cut-off result of the ROC curve analysis. Interpreting the ICE for IG% was much more intuitive and heterogeneous relationships were easily found. Similar to the pattern of the centered line, most patients showed little change in sepsis predictability up to an IG% of 4%. Since CRP also showed good predictability in the single-predictor PDP and was a significant factor along with IG% in the ROC and logistic regression analyses, we used a multi-predictor PDP to show the effect of the interaction between CRP and IG% on sepsis predictability. On this plot, when the IG% increased above 6% and the CRP level was above 160 mg/L, the probability of sepsis increased significantly, above 0.4. Considering that none of the biomarkers assessed individually exceeded 0.4 for predictability, the application of the IG% with CRP could significantly improve predictability.

Previous studies have reported on different aspects of the IG% in relation to sepsis. Karon et al. [18] reported that the IG% and IG count were of limited utility in predicting sepsis in a study of patients admitted to the emergency department. Additionally, unlike the findings of our study, Buoro et al. [19] reported that an IG < 10.0% was a valid cut-off for the diagnosis of sepsis in ICU patients. Ayres et al. [20] reported that an IG% < 2.0% was adequate for excluding sepsis as a diagnosis. However, these previous studies included patients who had various underlying etiologies, while only patients with severe burns who were admitted to the BICU at a burn specialized center were included in our study to identify the discriminatory power of IG% for patients with sepsis.

A few studies have been conducted on sepsis biomarkers in burn patients. Wineberg et al. [21] and Kundes et al. [22] studied procalcitonin in adults and children, respectively. In these studies, procalcitonin was shown to be a good marker for diagnosing sepsis in burn patients, but it was not effective in our study. The association between lactate and sepsis has also been assessed in several studies [23–25]. However, none of these studies have assessed sepsis in only burn patients. In our study, lactate appeared to have moderate discriminatory power according to the AUC, but the PDP analysis showed unclear predictability.

The advantage of using IG% is that it can be obtained from a routine complete blood count, and therefore does not require further laboratory protocols, while other biomarkers require additional time, procedures, and costs. Another advantage is that the IG% tends to be high in sepsis regardless of the WBC count. Patients with sepsis may not have an increase in neutrophils, and may even be neutropenic. In these situations, an increase in the IG% can be useful for identifying an acute infection, even when not suspected [5, 26]. Our results showed that the IG% was higher in the sepsis group, even when the WBC count was low or normal. It may therefore serve as an independent marker that can be used in cases of sepsis showing leukocytopenia.

Our study was unique in the following aspects: (1) the relationship between parameters and predictability was identified by applying a machine learning approach, and these visualized results increased reliability and allowed for us to review findings from different aspects; (2) the study was performed at a single center and only severe burn patients in a BICU were included, which helped minimize possible institutional or instrument-dependent biases; (3) our study provided an IG% cut-off value using a ROC curve analysis as well as machine learning technique to demonstrate its clinical applicability as an independent indicator of sepsis; and (4) a cut-off value for the CRP level and IG% were determined, increasing the co-utility of IG% and as a sepsis biomarker.

Our study also has several limitations. The number of patients involved was small. There was also a difference in the type of burns and %TBSA, which could have affected the distribution of the granulocyte indices. In addition, it would be difficult to apply our results to all age groups, as newborns have a higher IG% than adults [6]. Finally, the discriminatory power and the predictability of the IG% were not high, so it is insufficient to be used as a stand-alone biomarker for sepsis. CRP is a well-known, widely studied biomarker in sepsis. It showed better discriminatory power and the predictability than IG% in our study. As shown by multivariate ROC and PDP results, discriminatory power and the predictability were higher when CRP and IG% are combined than each alone. Therefore, IG% results can be useful when assessed as an auxiliary to CRP.

## Conclusions

In conclusion, IG% is moderately useful for predicting sepsis in patients with severe burns. We used a machine learning approach to predicting, observed an increase in the probability of developing sepsis when IG and CRP were higher than 6% and 160 mg/L, respectively. Our results emphasize that the IG% is useful as an auxiliary marker, can be applied quickly and easily without requiring additional procedures or costs to assist in the prevention of sepsis progression in patients with severe burns who have life-threatening conditions.

## Supplementary Information

**Additional file 1.** **Supplementary Figure 1.** Schematic diagram of the machine learning process for prediction of sepsis task.

## Data Availability

All data generated or analyzed during this study are included in this published article.

## Abbreviations

SIRS: Systemic inflammatory response syndrome
IG: Immature granulocytes
BICU: Burn intensive care unit
TBSA: Total body surface area
CRP: C-reactive protein
PDP: Partial dependence plot
ICE: Individual conditional expectation
SAPS-3: Simplified Acute Physiology Score-3
CRAB: Carbapenem-resistant *Acinetobacter baumannii*
CRPA: Carbapenem-resistant *Pseudomonas aeruginosa*
MRSA: Methicillin-resistant *Staphylococcus aureus*
