# Supplementary material for: Immature granulocyte percentage for prediction of sepsis in severe burn patients: a machine leaning-based approach
Source: BMC Infect Dis. 2021 Dec 16;21:1258. doi: 10.1186/s12879-021-06971-2 (PMC8680375; doi:10.1186/s12879-021-06971-2)
Supplement: Supplementary file 1 — Additional file 1. Supplementary Figure 1. Schematic diagram of the machine learning process for prediction of sepsis task. [file 12879_2021_6971_MOESM1_ESM.docx]

**Supplementary information**

**
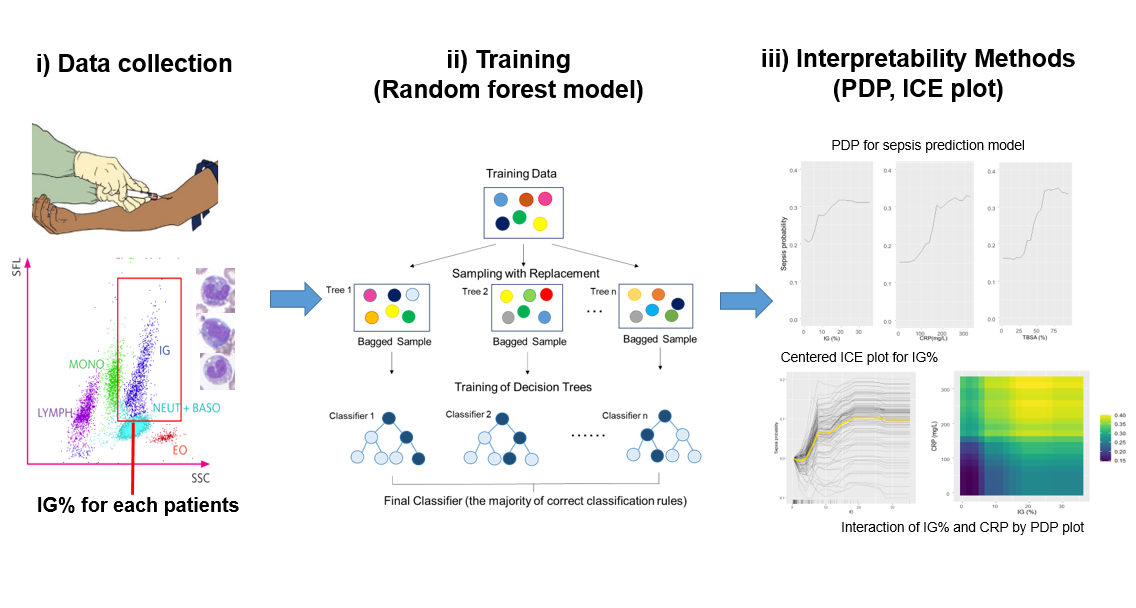
**

**Supplementary Figure 1. Schematic diagram of the machine learning process for prediction of sepsis task.** After data collection from each patient and training utilizing random forest model, partial dependence plots (PDP) and individual conditional expectations (ICE) are used to interpret the probability of sepsis. The PDP plot shows the marginal effect and interactions of IG% and other biomarkers for sepsis probability of machine learning model.
